# Supplementary material for: Evolutionary Patterns of the Chloroplast Genome in Vanilloid Orchids (Vanilloideae, Orchidaceae)
Source: Int J Mol Sci. 2023 Feb 14;24(4):3808. doi: 10.3390/ijms24043808 (PMC9966724; doi:10.3390/ijms24043808)
Supplement: Supplementary file 1 [file ijms-24-03808-s001.zip › ijms-2198085-supplementary.pdf]

Supplementary Tables S1-S3.

Table S1. The NGS results of six newly decoded Vanilloideae species.

**Scientific Name**	**NGS Method**	**# of raw reads**	**# of trimmed reads**	**Coverage**	**Voucher specimen and/or DNA number**
Lecanorchis japonica	MiSeq	10,228,582	9,994,252	76.9	PDBK2018-0250
Lecanorchis kiusiana	MiSeq	9,688,246	9,458,470	128.9	PDBK2018-0249
Pogonia japonica	MiSeq	21,314,324	21,084,838	464.6	PDBK2015-1272
Pogonia minor	MiSeq	9,508,082	9,378,346	563.2	PDBK2011-1673
Vanilla madagascariensis	HiSeq	64,556,822	64,556,822	4394	PDBKTMA2013-1861
Vanilla planifolia	HiSeq	16,568,124	16,568,124	592	PDBKTMA2013-1860

Table S2. Log-likelihood ratio (LRT) test result among eight regions under position effect and twenty remaining regions in mycoheterotrophic species. Regions with significant p-values (p-value < 0.05) are highlighted in red color.

**Category**	**Region**	**p-value**
Position effect	*ccs*A	0.48814321
Position effect	*rpl*2	0.032521691
Position effect	*rpl*22	0.990540247
Position effect	*rps*12 3'	0.21771596
Position effect	*rps*15	0.075109711
Position effect	*rps*19	0.982254224
Position effect	*rps*7	0.998829689
Position effect	*ycf*1	7.57649E-11
Mycoheterotrophy	*clp*P	0.019750885
Mycoheterotrophy	*inf*A	0.03997608
Mycoheterotrophy	*mat*K	0.395834929
Mycoheterotrophy	*rpl*2	0.233440934
Mycoheterotrophy	*rpl*14	0.99721688
Mycoheterotrophy	*rpl*16	0.197039906
Mycoheterotrophy	*rpl*20	0.684008455
Mycoheterotrophy	*rpl*36	0.188353267
Mycoheterotrophy	*rps*2	0.250458761
Mycoheterotrophy	*rps*3	0.003294773
Mycoheterotrophy	*rps*4	0.778982265
Mycoheterotrophy	*rps*7	0.169622992
Mycoheterotrophy	*rps*8	0.357812562
Mycoheterotrophy	*rps*11	0.440046445
Mycoheterotrophy	*rps*14	0.152648254
Mycoheterotrophy	*rps*18	0.000922093
Mycoheterotrophy	*rps*19	0.881219091
Mycoheterotrophy	*rps*12 5'	0.157522811
Mycoheterotrophy	*rps*12 3'	0.287089548
Mycoheterotrophy	*ycf*2	0.090170767

Table S3. Used sequence information in this study. Red scientific name indicates that the species' NGS data was generated in this study.

Scientific Name	NCBI Accession Number (Plastome)	Length (bp)	LSC (bp)	SSC (bp)	IR (bp)	GC (%)	Coverage (x)	Subfamily	Tribe
Bletilla ochracea	NC029483	157,431	85,810	17,949	26,836	37.3	-	Epidendroideae	Arethuseae
Bletilla striata	NC028422	157,393	86,213	17,742	26,719	37.2	-	Epidendroideae	Arethuseae
Calanthe davidii	NC037438	153,629	86,045	15,672	25,956	36.9	-	Epidendroideae	Collabieae
Calanthe triplicata	NC024544	158,759	87,305	18,460	26,497	36.7	-	Epidendroideae	Collabieae
Cymbidium lancifolium	NC029712	149,945	84,716	13,895	25,667	37.1	-	Epidendroideae	Cymbidieae
Cymbidium macrorhizon	KY354040	149,859	85,187	13,766	25,453	37	-	Epidendroideae	Cymbidieae
Cymbidium sinense	NC021430	155,548	84,920	17,666	26,481	37	-	Epidendroideae	Cymbidieae
Erycina pusilla	NC018114	143,164	83,733	11,675	23,878	36.7	-	Epidendroideae	Cymbidieae
Eulophia zollingeri	NC037212	145,201	81,566	13,091	25,272	36.9	-	Epidendroideae	Cymbidieae
Oncidium sphacelatum	NC028148	147,761	83,575	12,670	25,758	37.1	-	Epidendroideae	Cymbidieae
Cattleya crispata	NC026568	148,343	85,973	13,261	24,495	37.3	-	Epidendroideae	Epidendreae
Cattleya liliputana	NC032083	147,092	85,945	13,149	24,304	37.4	-	Epidendroideae	Epidendreae
Corallorhiza bulbosa	NC025659	148,643	82,851	12,368	26,712	37.1	-	Epidendroideae	Epidendreae
Corallorhiza macrantha	NC025660	151,031	84,263	12,544	27,112	37.2	-	Epidendroideae	Epidendreae
Corallorhiza maculata var. *maculata*	KM390014	146,886	80,395	12,885	26,803	36.8	-	Epidendroideae	Epidendreae
Corallorhiza maculata var. *mexicana*	KM390015	151,506	84,347	12,671	27,244	37.1	-	Epidendroideae	Epidendreae
Corallorhiza maculata var. *occidentalis*	KM390016	146,595	81,363	12,368	26,432	36.9	-	Epidendroideae	Epidendreae
Corallorhiza mertensiana	NC025661	147,941	81,109	13,774	26,529	36.8	-	Epidendroideae	Epidendreae
Corallorhiza odontorhiza	NC025664	147,317	82,257	13,508	25,776	37	-	Epidendroideae	Epidendreae

Corallorhiza striata var. *vreelandii*	JX087681	137,505	72,631	12,388	26,243	36.4	-	Epidendroideae	Epidendreae
Corallorhiza trifida	NC025662	149,384	83,092	14,420	25,936	37.2	-	Epidendroideae	Epidendreae
Corallorhiza wisteriana	NC025663	146,437	76,350	17,743	26,172	37.1	-	Epidendroideae	Epidendreae
Masdevallia coccinea	NC026541	157,423	84,957	18,448	27,009	36.8	-	Epidendroideae	Epidendreae
Masdevallia picturata	NC026777	156,045	84,948	18,029	26,534	36.9	-	Epidendroideae	Epidendreae
Gastrodia elata	NC037409	35,304	(SC Only)	(SC Only)	-	34.2	-	Epidendroideae	Gastrodieae
Dendrobium nobile	NC029456	153,660	85,686	14,654	26,660	37.5	-	Epidendroideae	Malaxideae
Dendrobium officinale	NC024019	152,221	85,109	14,516	26,298	37.5	-	Epidendroideae	Malaxideae
Oberonia japonica	NC035832	142,996	81,669	10,969	25,179	37.4	-	Epidendroideae	Malaxideae
Aphyllorchis montana	NC030703	94,559	(SC Only)	(SC Only)	-	37.1	-	Epidendroideae	Neottieae
Cephalanthera humilis	NC030706	157,011	86,908	15,133	27,485	37.3	-	Epidendroideae	Neottieae
Cephalanthera longifolia	NC030704	161,877	88,806	19,187	26,942	37.2	-	Epidendroideae	Neottieae
Epipactis mairei	NC030705	159,019	86,377	18,816	26,913	37.3	-	Epidendroideae	Neottieae
Epipactis veratrifolia	NC030708	159,719	87,043	18,854	26,911	37.3	-	Epidendroideae	Neottieae
Neottia acuminata	NC030709	83,190	51,145	5,371	13,337	36.6	-	Epidendroideae	Neottieae
Neottia camtschatea	NC030707	106,385	52,960	9,273	22,076	37.2	-	Epidendroideae	Neottieae
Neottia fugongensis	NC030711	156,536	85,357	18,311	26,434	31.6	-	Epidendroideae	Neottieae
Neottia listeroides	NC030713	110,246	45,021	9,597	27,814	37.2	-	Epidendroideae	Neottieae
Neottia nidus-avis	NC016471	92,060	36,422	7,822	23,908	34.4	-	Epidendroideae	Neottieae
Neottia ovata	NC030712	156,978	85,433	18,071	26,737	37.6	-	Epidendroideae	Neottieae
Neottia pinetorum	NC030710	155,959	84,449	18,104	26,703	37.5	-	Epidendroideae	Neottieae
Epipogium aphyllum	NC026449	30,650	8,030	-	11,310	32.8	-	Epidendroideae	Nervilieae
Epipogium roseum	NC026448	19,047	9,618	8,907	261	30.6	-	Epidendroideae	Nervilieae
Elleanthus sodiroi	NC027266	161,511	88,425	18,880	27,103	37.1	-	Epidendroideae	Sobralieae

Sobralia callosa	NC028147	161,430	88,666	18,794	26,985	37.1	-	Epidendroideae	Sobralieae
Gastrochilus fuscopunctatus	KX871233	146,183	83,125	11,146	25,956	36.8	-	Epidendroideae	Vandeae
Gastrochilus japonicus	KX871236	147,697	84,695	11,174	25,914	36.8	-	Epidendroideae	Vandeae
Neofinetia cultivar	KT726907	146,497	83,808	11,775	25,457	36.6	-	Epidendroideae	Vandeae
Neofinetia falcata	KT726909	146,491	83,802	11,775	25,457	36.6	-	Epidendroideae	Vandeae
Neofinetia richardsiana	KT726908	146,498	83,809	11,775	25,457	36.6	-	Epidendroideae	Vandeae
Pelatantheria scolopendrifolia	KX871232	146,860	86,075	11,735	24,525	36.5	-	Epidendroideae	Vandeae
Phalaenopsis aphrodite subsp. *formosana*	AY916449	148,964	85,957	11,543	25,732	36.7	-	Epidendroideae	Vandeae
Phalaenopsis equestris	JF719062	148,959	85,967	11,300	25,846	36.7	-	Epidendroideae	Vandeae
Phalaenopsis hybrid cultivar	KJ944326	148,918	85,885	11,523	25,755	36.7	-	Epidendroideae	Vandeae
Phalaenopsis pulcherrima	MG459020	149,272	85,749	11,653	25,935	36.8	-	Epidendroideae	Vandeae
Thrixspermum japonicum	KX871234	149,220	85,301	11,546	26,187	36.1	-	Epidendroideae	Vandeae
Goodyera fumata	NC026773	155,643	84,077	18,342	26,612	37.3	-	Orchidoideae	Cranichideae
Goodyera procera	NC029363	153,240	82,032	18,406	26,401	37.6	-	Orchidoideae	Cranichideae
Goodyera schlechtendaliana	NC029364	154,348	83,215	18,051	26,541	37.2	-	Orchidoideae	Cranichideae
Goodyera velutina	NC029365	152,692	82,443	17,247	26,501	36.9	-	Orchidoideae	Cranichideae
Kuhlhasseltia nakaiana	KY354041	147,614	81,617	13,673	26,162	39.5	-	Orchidoideae	Cranichideae
Ludisia discolor	NC030540	153,054	82,675	17,233	26,573	37	-	Orchidoideae	Cranichideae
Rhizanthella gardneri	NC014874	59,190	26,360	13,295	9,767	34.2	-	Orchidoideae	Diurideae
Habenaria pantlingiana	NC026775	153,951	83,641	17,370	26,470	36.6	-	Orchidoideae	Orchideae
Habenaria radiata	NC035834	155,353	84,833	17,718	26,401	36.5	-	Orchidoideae	Orchideae
Cypripedium formosanum	NC026772	178,131	102,188	21,921	27,011	33.9	-	Cypripedioideae	Cypripedioideae
Cypripedium japonicum	NC027227	174,417	97,322	21,911	27,592	34.5	-	Cypripedioideae	Cypripedioideae
Paphiopedilum armeniacum	NC026779	162,682	91,734	3,666	33,641	35.4	-	Cypripedioideae	Cypripedioideae
Paphiopedilum dianthum	NC036958	154,699	86,861	2,416	32,711	35.9	-	Cypripedioideae	Cypripedioideae

Paphiopedilum niveum	NC026776	159,108	89,856	5,194	32,029	35.7	-	Cypripedioideae	Cypripedioideae
Phragmipedium longifolium	NC028149	151,157	88,367	13,066	24,862	36.1	-	Cypripedioideae	Cypripedioideae
Pogonia japonica	MN200371	158,200	87,447	5,387	32,683	36.4	464.6	Vanilloideae	Pogonieae
Pogonia minor	MN200372	158,170	87,457	5,375	32,669	36.4	563.2	Vanilloideae	Pogonieae
Cyrtosia septentrionalis	MH615835	96,859	58,085	10,414	17,946	34.8	-	Vanilloideae	Vanilleae
Lecanorchis japonica	MN200364	70,498	28,197	14,493	13,904	30.4	76.9	Vanilloideae	Vanilleae
Lecanorchis kiusiana	MN200363	74,084	30,824	14,118	14,571	30	128.9	Vanilloideae	Vanilleae
Vanilla aphylla	NC035320	150,184	87,379	2,131	30,337	35	-	Vanilloideae	Vanilleae
Vanilla madagascariensis	MN200374	151,552	87,490	1,254	31,404	34.6	4,394.0	Vanilloideae	Vanilleae
Vanilla planifolia 1	MN200375	147,714	86,061	2,037	29,808	35.4	592.0	Vanilloideae	Vanilleae
Vanilla planifolia 2	NC026778	148,011	86,358	2,037	29,808	35.4	-	Vanilloideae	Vanilleae
Vanilla pompona	NC036809	148,009	86,358	2,037	29,807	35.4	-	Vanilloideae	Vanilleae
Apostasia odorata	NC030722	159,285	86,172	18,765	27,174	35.7	-	Apostasioideae	Apostasioideae
Apostasia wallichii	NC036260	156,126	83,031	20,187	26,454	36.1	-	Apostasioideae	Apostasioideae
Neuwiedia singapureana	KM244735	162,174	89,657	18,399	27,059	35.8	-	Apostasioideae	Apostasioideae
Neuwiedia zollingeri var. *singapureana*	LC199503	161,068	88,910	18,056	27,051	36	-	Apostasioideae	Apostasioideae
Allium cepa	KM088013	153,529	82,662	17,931	26,468	36.8	-	Amarylidaceae (Outgroup)	Amarylidaceae (Outgroup)
Eustrephus latifolius	NC025305	159,736	82,403	13,607	31,863	38.1	-	Asparagaceae (Outgroup)	Asparagaceae (Outgroup)
Iris gatesii	NC024936	153,441	82,702	18,371	26,184	37.9	-	Iridaceae (Outgroup)	Iridaceae (Outgroup)
Iris sanguinea	NC029227	152,408	82,340	18,016	26,026	38	-	Iridaceae (Outgroup)	Iridaceae (Outgroup)
Fritillaria hupehensis	NC024736	152,145	81,894	17,553	26,349	37	-	Liliales (Outgroup)	Liliales (Outgroup)

2

Supplementary Figures S1-S4.

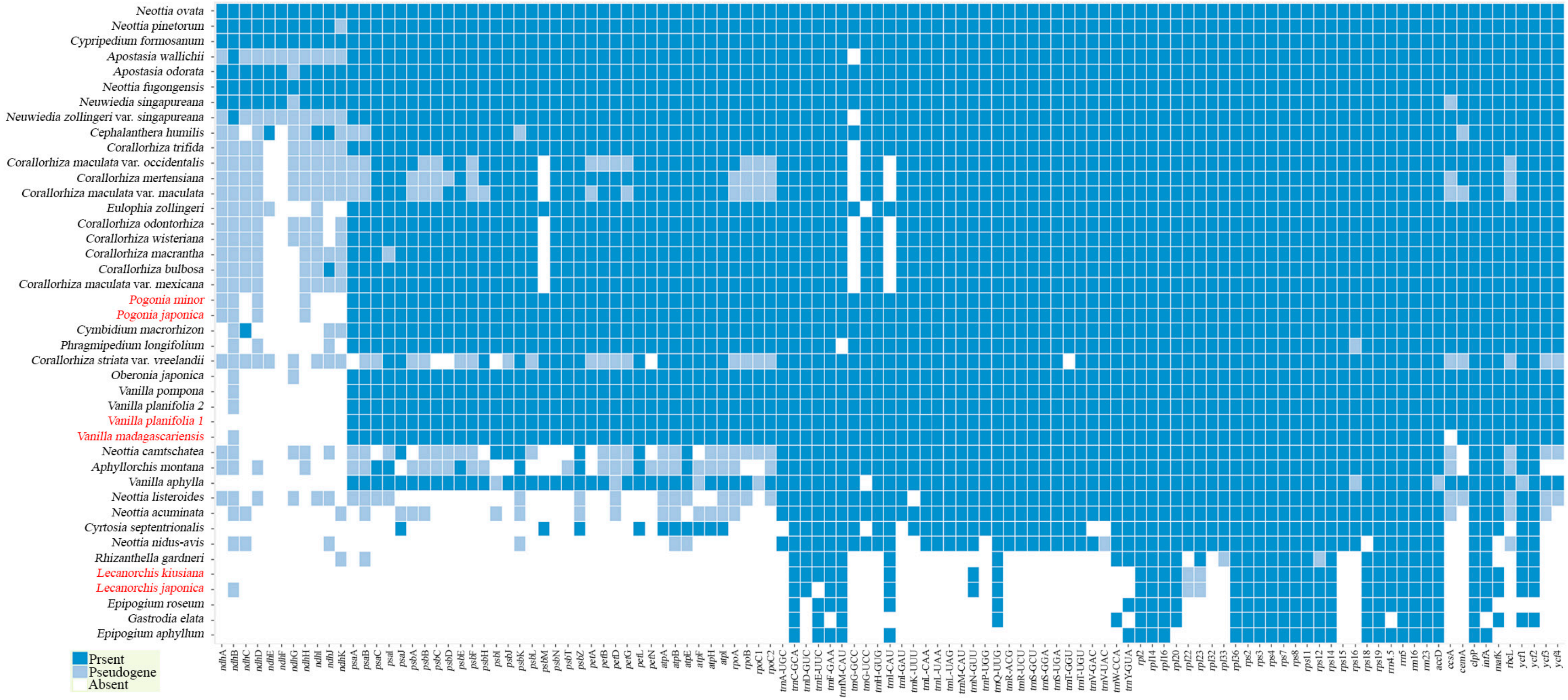

Figure S1. Gene contents heatmap of Orchidaceae, including vanilloid species.

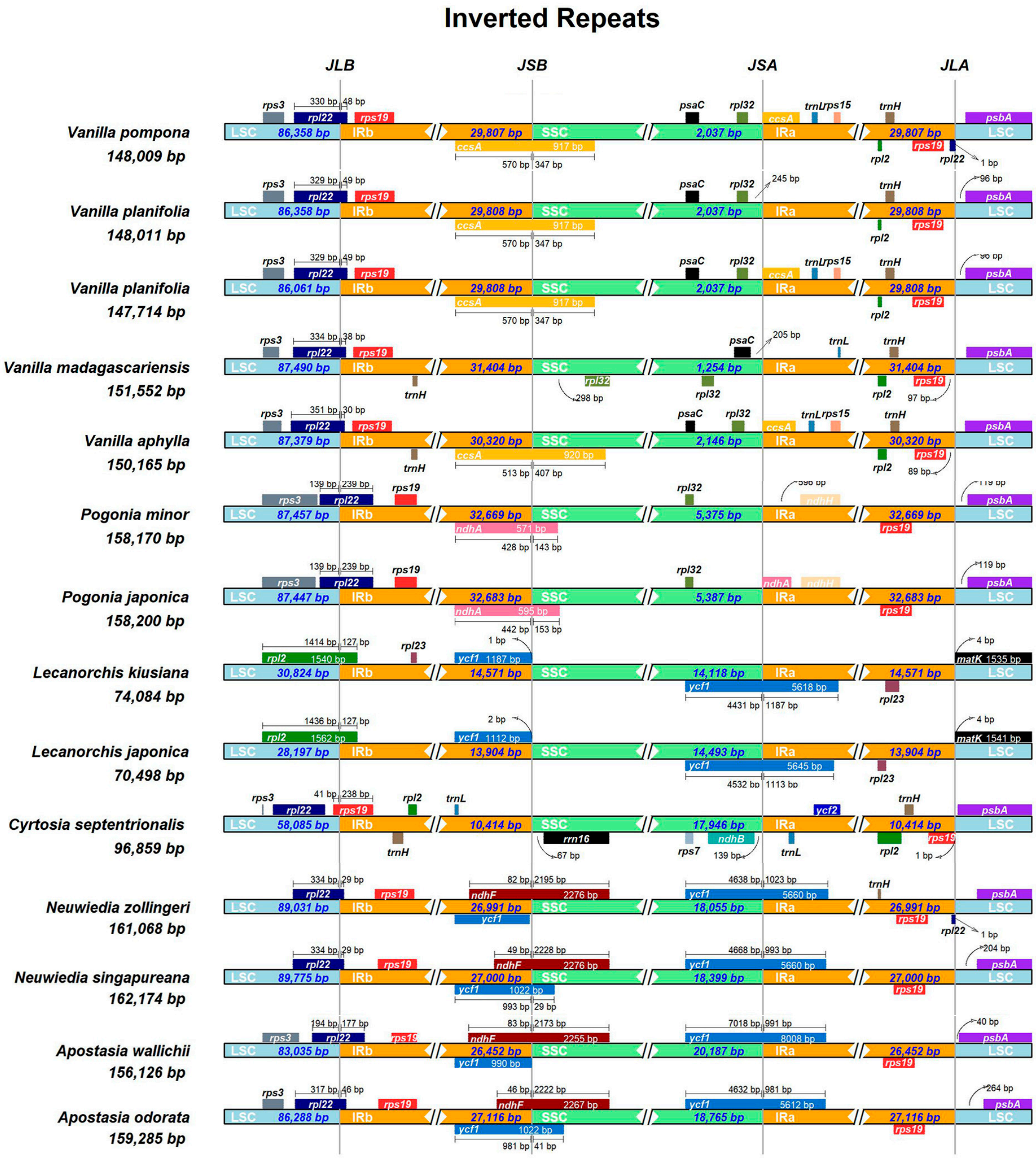

Figure S2. IR junctions of ten Vanilloideae and four Apostasioideae species.

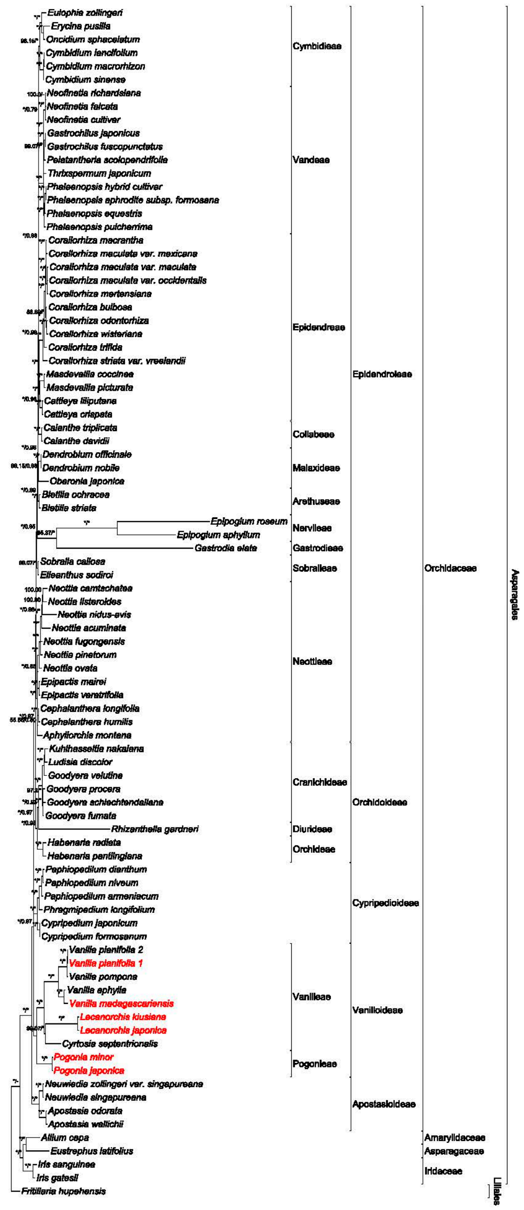

Figure S3. A maximum likelihood (ML) tree inferred from 89 species of Orchidaceae. The nucleotide sequences of 79 protein-coding genes and four rRNA genes were aligned individually and concatenated to be a single matrix.

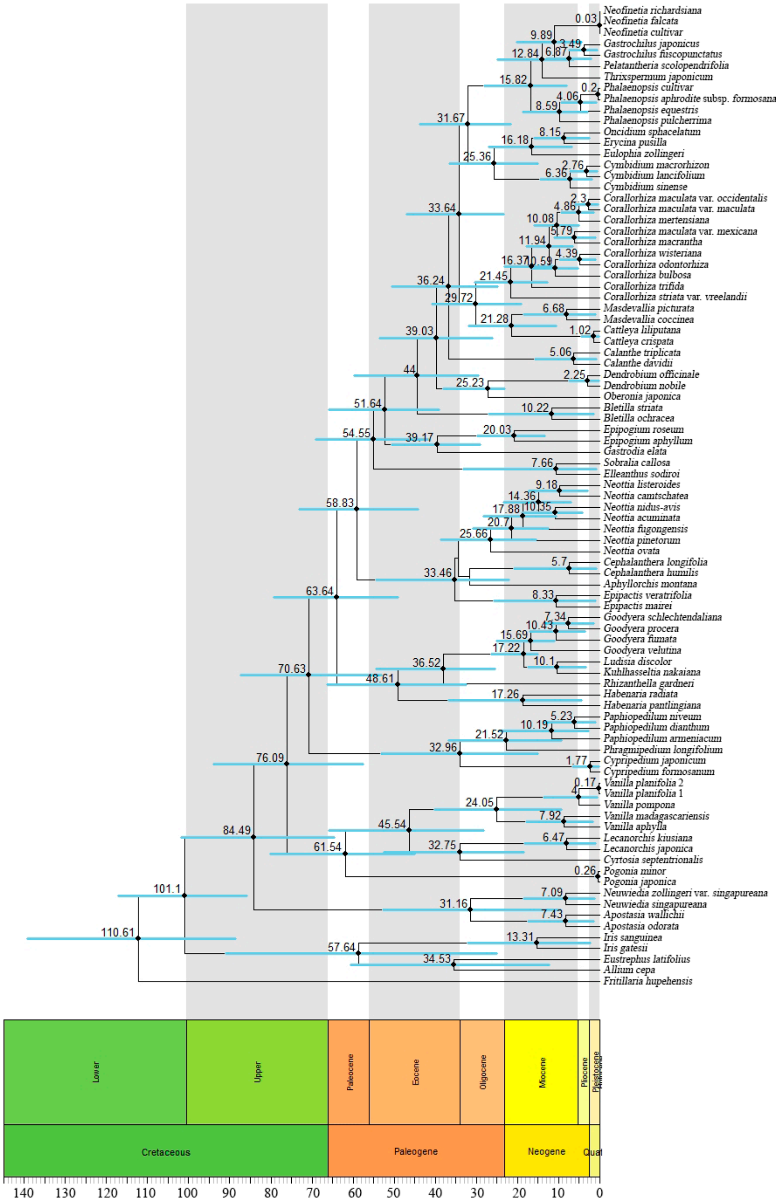

Figure S4. Divergence time estimation of Orchidaceae.
